# The Elmira Eye and Ear Institute

**Published:** 1870-01

**Authors:** Theo. N. Lescher

**Affiliations:** Registrar


### The Elmira Eye and Ear Institute.

For the treatment of all diseases of the eye and ear. Open from 8 a.m. to 5 p.m.

THAD. S. UP DE GRAFF, M. D.,
Surgeon.

REPORT OF CASES.—Under treatment in the Elmira Eye & Ear Institute, for the Quarter ending January 1st, 1870:

| | |
|---|---|
| Under treatment for Granular Lid | 8 |
| " " " Scrofulous Ophthalmia, | 5 |
| " " " Gonorrhœal " | 4 |
| " " " Purulent " of Infants | 1 |
| " " " Syphilitic Iritis, | 2 |
| " " " Pannus, | 4 |
| " " " Rheumatic Ophthalmia, | 1 |
| " " " Glaucoma, | 3 |
| " " " Catarrhal Ophthalmia | 5 |
| " " " Simple Conjunctivitis, | 2 |
| " " " Suppurative Keratitis, | 3 |
| " " " Meibomitis | 2 |
| " " " Astigmatism | 1 |
| " " " Opacity of Cornea, | 3 |
| " " " Incised wound of Cornea, | 3 |
| " " " Otorrhœa, | 6 |
| " " " Deafness, | 5 |
| " " " Catarrh and Disease of Throat | 6 |
| " " " Spermatorrhœa, | 18 |
| Operated upon for Cataract, | 7 |
| " " " Iridectomy, | 4 |
| " " " Corneal Staphyloma | 1 |
| " " " Entropium, | 2 |
| " " " Extirpation of Eye, | 1 |
| " " " Pterygium, | 4 |
| " " " Strabismus, (Cross Eye.) | 6 |
| " " " Obstruction Lachrymal Duct, | 2 |
| " " " Club Foot | 2 |
| " " " Removal of Tumors, | 4 |
| " " " Hœmorrhoids | 2 |
| " " " Nasal Polypii, | 3 |
| " " " Lacerated Perinæum, | 1 |
| " " " Prolapsus Ani, | 1 |
| " " " Hydrocile, | 1 |
| " " " Aural Polypii, | 3 |
| " " " Cancer of Hand, | 1 |
| " " " Stricture of Throat, | 1 |
| " " " Amputation of Cervix Uteri, | 1 |
| Insertion of Artificial Eyes, | 4 |
| Minor Surgical Operations, | 8 |
| Total number of Cases, | 141 |

THEO. N. LESCHER, Registrar.

NOTICE TO CITY SUBSCRIBERS.—The BISTOURY will be delivered to business places in the city, by a carrier. Should any of our subscribers fail to get their number, they will please report the same to this office.

NOTICE TO ADVERTISERS.—Those who intend discontinuing their cards with this volume, will please notify us at once, so that we can supply their places before next issue.

